# BMP signalling in human fetal ovary somatic cells is modulated in a gene-specific fashion by GREM1 and GREM2

**DOI:** 10.1093/molehr/gaw044

**Published:** 2016-09-06

**Authors:** Rosemary A. Bayne, Douglas J. Donnachie, Hazel L. Kinnell, Andrew J. Childs, Richard A. Anderson

**Affiliations:** 1 MRC Centre for Reproductive Health, University of Edinburgh, Queen's Medical Research Institute, EdinburghEH16 4TJ, UK; 2 Department of Comparative Biomedical Sciences, The Royal Veterinary College, LondonNW1 0TU, UK

**Keywords:** BMP, BMP antagonist, human fetal ovary, ovarian somatic cell, pre-granulosa cell, ovarian development

## Abstract

**STUDY QUESTION:**

Do changes in the expression of bone morphogenetic proteins (BMPs) 2 and 4, and their antagonists Gremlin1 (GREM1) and Gremlin2 (GREM2) during human fetal ovarian development impact on BMP pathway activity and lead to changes in gene expression that may influence the fate and/or function of ovarian somatic cells?

**STUDY FINDING:**

BMPs 2 and 4 differentially regulate gene expression in cultured human fetal ovarian somatic cells. Expression of some, but not all BMP target genes is antagonised by GREM1 and GREM2, indicating the existence of a mechanism to fine-tune BMP signal intensity in the ovary. Leucine-rich repeat-containing G-protein coupled receptor 5 (*LGR5)*, a marker of immature ovarian somatic cells, is identified as a novel transcriptional target of BMP4.

**WHAT IS KNOWN ALREADY:**

Extensive re-organisation of the germ and somatic cell populations in the feto-neonatal ovary culminates in the formation of primordial follicles, which provide the basis for a female's future fertility. BMP growth factors play important roles at many stages of ovarian development and function. GREM1, an extracellular antagonist of BMP signalling, regulates the timing of primordial follicle formation in the mouse ovary, and mRNA levels of *BMP4* decrease while those of *BMP2* increase prior to follicle formation in the human fetal ovary.

**STUDY DESIGN, SAMPLES/MATERIALS, METHODS:**

Expression of genes encoding BMP pathway components, BMP antagonists and markers of ovarian somatic cells were determined by quantitative (q)RT-PCR in human fetal ovaries (from 8 to 21 weeks gestation) and fetal ovary-derived somatic cell cultures. Ovarian expression of GREM1 protein was confirmed by immunoblotting. Primary human fetal ovarian somatic cell cultures were derived from disaggregated ovaries by differential adhesion and cultured in the presence of recombinant human BMP2 or BMP4, with or without the addition of GREM1 or GREM2.

**MAIN RESULTS AND THE ROLE OF CHANCE:**

We demonstrate that the expression of BMP antagonists *GREM1*, *GREM2* and *CHRD*  increases in the lead-up to primordial follicle formation in the human fetal ovary, and that the BMP pathway is active in cultured ovarian somatic cells. This leads to differential changes in the expression of a number of genes, some of which are further modulated by GREM1 and/or GREM2. The positive transcriptional regulation of *LGR5* (a marker of less differentiated somatic cells) by BMP4 *in vitro* suggests that increasing levels of GREM1 and reduced levels of BMP4 as the ovary develops *in vivo* may act to reduce LGR5 levels and allow pre-granulosa cell differentiation.

**LIMITATIONS, REASONS FOR CAUTION:**

While we have demonstrated that markers of different somatic cell types are expressed in the cultured ovarian somatic cells, their proportions may not represent the same cells in the intact ovary which also contains germ cells.

**WIDER IMPLICATIONS OF THE FINDINGS:**

This study extends previous work identifying germ cells as targets of ovarian BMP signalling, and suggests BMPs may regulate the development of both germ and somatic cells in the developing ovary around the time of follicle formation.

**LARGE SCALE DATA:**

Not applicable.

**STUDY FUNDING/COMPETING INTERESTS:**

This work was supported by The UK Medical Research Council (Grant No.: G1100357 to RAA), and Medical Research Scotland (Grant No. 345FRG to AJC). The authors have no competing interests to declare.

## Introduction

Female reproductive lifespan is determined before birth, with the formation of a pool of primordial follicles within the fetal ovaries, which constitute the supply of eggs for her entire life. In mammals, primordial follicle formation results from a highly dynamic period of germ and somatic cell reorganisation within the ovary. This occurs during fetal life in humans, and around the time of birth in mice. The size of the primordial follicle pool is determined by a mixture of germ cell proliferation and apoptosis, and interactions with surrounding somatic cells (Findlay *et al*., 2015), mediated by juxtacrine, paracrine and endocrine factors within the ovary. In mice, follicle formation occurs in two distinct waves (Mork *et al*., 2012, Zheng *et al*., 2014), with an early population of follicles assembled in the medulla which support puberty and the early phases of fertility, and second cohort formed in the cortex, which supports long-term female fertility.

The somatic cells which contribute to these different follicular cohorts also appear to be distinct. Somatic cells expressing the transcription factor Foxl2 are found in the medullary region, and form the pre-granulosa cells of the first wave of medullary primordial follicles (Mork *et al*., 2012; Zheng *et al*., 2014). Leucine-rich repeat-containing G-protein coupled receptor 5 (LGR5; a known marker of adult stem cells (Hirsch *et al*., 2014, Ng *et al*., 2014)) is expressed by somatic cells in the cortical region of fetal and postnatal mouse ovary, and LGR5-positive cells appear to be the progenitors of the pre-granulosa cells of follicles which form in the ovarian cortex (Rastetter *et al*., 2014). A third population of cells, expressing nuclear receptor subfamily 2 group F member 2 (NR2F2) (also known as COUP transcription factor 2, COUPTFII), may be of mesenchymal origin and contribute to the theca cell population as follicles grow (Rastetter *et al*., 2014), and are possibly equivalent to vascular associated somatic cells labelled by transcription factor MafB (MAFB) (Maatouk *et al*., 2012). The intrinsic and extrinsic mechanisms governing the specification and maintenance of these different populations of somatic cells remains to be established, however their distinct spatial distributions may support a role for localised growth factor signalling in regulating their ontogeny.

Bone Morphogenetic Proteins (BMPs) are critical regulators of ovarian development and function. BMP4 is required for primordial germ cell (PGC) specification, migration and maintenance (Pangas, 2012), and may contribute to the regulation of follicle growth. Bmp2 expression is restricted to the fetal mouse ovary after sex determination, and its expression is driven by WNT signalling (Yao *et al*., 2004). BMP2 expression was also detected in the somatic cells of the developing hamster ovary around the time of follicle formation (Chakraborty and Roy, 2015), and culture of feto-neonatal hamster ovaries with recombinant BMP2 reduces apoptosis and promotes primordial follicle formation, possibly by promoting the transition of mitotic germ cells into meiotic oocytes (Chakraborty and Roy, 2015). In humans, levels of BMP4 transcripts fall across gestation as germ cells enter meiosis, while levels of BMP2 rise (Childs *et al*., 2010), and culture of human fetal ovaries with BMP4-induced germ cell apoptosis (Childs *et al*., 2010).

We have previously reported that BMP4 levels fall and BMP2 levels rise in the human fetal ovary with increasing gestation, while expression of the two Type I BMP receptors and their activated downstream mediator pSMAD1/5/8 appeared to be predominantly in germ cells. Weak staining for BMP receptor 1A (BMPR1A) was detected in the somatic cells of 9-week ovaries, however, suggesting these two may be possible cellular targets of BMP activity (Childs *et al*., 2010). Whether the BMP pathway is active in human ovarian somatic cells has yet to be explored.

BMP pathway activity is regulated at a number of levels. Extracellular antagonists, including members of the DAN/Cerberus family (Walsh *et al*., 2010), bind BMPs and prevent them from associating with their receptors. These include GREMLIN (GREM1), its closely related homologue GREM2 (also called PRDC) as well as NOGGIN (NOG) and CHORDIN (CHRD). Disruption of Grem1 in mice leads to reduced oocyte numbers, delayed meiotic progression as well as delayed primordial follicle formation at the neonatal stage (Myers *et al*., 2011), suggesting that limiting BMP activity is important for the timely assembly of primordial follicles. CHRD and NOG are expressed predominantly in the granulosa cell compartment of small and growing follicles in cattle, and *ex vivo* experiments suggest that they contribute to intra-follicular BMP and activin signalling (Glister *et al*., 2011). In addition to these extracellular regulators of BMP activity, intracellular inhibitory SMADs (I-SMADs, SMADs 6 and 7) act to block SMAD1/5/8 phosphorylation, and/or nuclear translocation. SMAD6 is strongly expressed in the oocytes of primordial follicles in the mouse, and only weakly expressed in growing follicles or somatic cells (Gueripel *et al*., 2004) but is expressed by somatic cells surrounding developing germ cells in the human fetal ovary (Childs *et al*., 2010).

While it is clear that BMP signalling is involved in human ovarian development, the potential implications of the apparent switch from a BMP4 to a BMP2-predominant environment and the presence and functional roles of BMP antagonists has not been explored. Here we examined the expression of a number of extracellular BMP antagonists in the human fetal ovary and utilised culture of primary human fetal ovary somatic cells to characterise the BMP pathway in these cells and examine the possible roles of GREM1 and GREM2 in regulating this pathway. To determine whether BMPs may play a role in the specification or development of ovarian somatic cells, we also examined whether expression of somatic cell markers LGR5 and FOXL2 were modulated by BMPs or GREM1/2 in the cultured somatic cells.

## Materials and Methods

### Collection of human fetal ovaries

Ovaries were obtained from morphologically normal human fetuses (8–21 weeks gestational age) following medical termination of pregnancy. Written informed consent was obtained in accordance with national guidelines, and with ethical approval from Lothian Research Ethics Committee (Study Code LREC 08/S1101/1). Gestational age was measured by ultrasound scan before the procedure and confirmed by measuring foot length afterwards. Ovaries were removed and either snap frozen and stored at −80°C for subsequent extraction of RNA or protein, or placed in Hanks Buffered Salt Solution (HBSS; Sigma-Aldrich, Dorset, UK) for disaggregation and isolation of ovarian somatic cells as described below. A total of 26 fetal ovary specimens and 6 primary ovary somatic cell cultures were used in this study.

### Derivation of primary human fetal ovary somatic cell cultures

Each ovary was cleaned of extraneous tissue in HBSS and then placed, one ovary at a time, onto a dimple slide with 50 µl of 10 mg/ml Collagenase Type IV (Sigma-Aldrich; C1889-50MG) in HBSS and pulled apart with 19G needles. The tissue fragments and liquid were transferred to a 1.5 ml tube and the slide washed with a further 100 µl of collagenase solution, which was pooled with the tissue suspension. This procedure was repeated with the second ovary, the tissue pooled with that of the first ovary, and combined with the remainder of the 500 µl collagenase stock. The tissue/collagenase mix was incubated in a Thermomixer (Eppendorf, Stevenage, UK) at 37°C for 10 min with regular pipetting to ensure complete disaggregation of the tissue. 50 µl of DNase I (7 mg/ml stock in HBSS; Sigma Aldrich; DN25-100MG) was added and incubation continued for a further 5 min at 37°C. The cell suspension was then spun down at 500 g for 5 min in a micro-centrifuge, and the pellet washed twice in 1 ml HBSS. After the second wash, the pellet was re-suspended in 1 ml of somatic cell culture medium (SCCM; Dulbecco's Modified Eagles Medium (DMEM; w/o phenol red)/10% Fetal Bovine Serum (FBS)/2 mM L-glutamine/1× MEM Non-Essential Amino Acids (NEAA)) with 1× penicillin/streptomycin/amphotericin (P/S/A) added (all Life Technologies, Paisley, UK). The suspension was filtered through a 70 µm filter in a 50 ml Falcon tube, transferred into a fresh 1.5 ml tube, spun down again and re-suspended in 1.2 ml SCCM. Two hundred microliter of the cells were transferred to another 1.5 ml tube, spun down, washed in Dulbecco's PBS (DPBS, Life Technologies) and re-suspended in 350 µl of RLT Buffer (Qiagen, Crawley, UK) containing 143 mM 2-mercaptoethanol (2-ME, Sigma-Aldrich) and stored at −80°C. These cells were used to make RNA (0 timepoint) for cell characterisation by qRT-PCR. The remaining cells were split over 2 wells of a 12-well plate, and each well made up to 1 ml with SCCM. Cells were incubated at 37°C/ 5% CO_2_ in air overnight to allow separation of somatic and germ cells by differential adhesion to the plate surface. Next day, culture supernatant containing germ cells, dead cells and other debris was removed and the adherent somatic cells washed three times in SCCM. This process was repeated again the next day to remove residual germ cells. Once cells were confluent, they were passaged by trypsinisation and re-plated in larger wells until sufficient to be placed in cell culture flasks. At this stage a portion of the cells were spun down and re-suspended in 1 ml Bambanker (Anachem, Beds., UK) for cryopreservation. A total of six cell cultures (Table I) were derived and used in this study and could be passaged at least 12 times. At each of the earlier passages, a small aliquot of cells was lysed in RLT+2-ME for RNA extraction in order to allow characterisation of expressed marker genes by qRT-PCR.

**Table I:** Human fetal ovarian somatic cell cultures.

Cell Culture	Gestation of Ovaries
FT2692	16 weeks + 1 day
FT3010	15 weeks + 1 day
FT3096	9 weeks + 2 days
FT3329	17 weeks + 5 days
FT3330	14 weeks + 5 days
FT3331	16 weeks + 1 day

### Treatment of primary human fetal ovarian somatic cell cultures with recombinant growth factors and antagonists

Human fetal ovarian somatic cells were cultured in SCCM and passaged until there were sufficient cells for each experiment. Cells for treatments were used at passages 5–10. Cells were plated in 24 or 12-well plates (for RNA extraction) or 6-well plates (for protein extraction) to give ~80% confluency the next day (approximately 1 × 10^5^ cells/cm^2^ of culture area), and were then washed three times in DPBS before starving in serum-free SCCM overnight. Treatments and appropriate vehicle controls (Table II) were prepared in serum-free SCCM and applied to the cells for the appropriate time (24 h for RNA extraction or 1 h for pSMAD phosphorylation determination).

**Table II:** Cell treatments.

Treatment	Source	Cat. No.	Vehicle	Stock Conc.	Working Conc.
Recombinant Human BMP4	Life Technologies (Paisley, UK)	PHC-9534	4 mM HCl/ 0.1% BSA in PBS	10 μg/ml	1–100 ng/ml as indicated in text
Recombinant Human BMP2	Life Technologies (Paisley, UK)	PHC-7145	4 mM HCl/ 0.1% BSA in PBS	100 μg/ml	1–100 ng/ml as indicated in text
Recombinant Human Gremlin, CF	R&D Systems (Abingdon, UK)	5190-GR-050	PBS	200 μg/ml	1 μg/ml
Recombinant Human PRDC/GREM2, CF	R&D Systems (Abingdon, UK)	8436-PR-050	4 mM HCl in PBS	500 μg/ml	100 ng/ml

### Extraction of RNA and first strand cDNA synthesis

RNA was extracted from frozen fetal ovaries using the RNeasy Mini Kit (Qiagen; specimens over 12 weeks) or RNeasy Micro Kit (Qiagen; specimens at 8–11 weeks and RLT lysates of treated cell extracts) with on-column DNase I digestion, according to the manufacturer's protocols. RNA concentration and purity was measured on the NanoDrop 1100 (NanoDrop Products, Wilmington, DE, USA) and first strand cDNA synthesised from 500 ng RNA (ovaries or cell treatments) or 200 ng RNA (early cell passages) using the Maxima First Strand cDNA Synthesis Kit (Fisher Scientific, Loughborough, UK) +/− the RT enzyme mix (RT+ and RT− respectively) according to the manufacturer's recommendations.

### QRT-PCR

Quantitative reverse transcriptase-PCR (qRT-PCR) was performed in 10 μl Reactions of 1× Brilliant III SYBR green Mastermix (Agilent Technologies, Stockport, UK) containing 500 nM each of relevant forward and reverse primers (Supplementary Table 1), 2 μl of a 1/20 dilution of each cDNA and 300 nM of passive Reference Dye (provided with the kit) for the ABI 7900HTFast Thermocycler (Life Technologies). Cycling conditions of 95°C for 3 min followed by 40 cycles of 95°C for 5 s and 60°C for 15 s were used, as recommended by the manufacturer. For each gene of interest, each RT(+) sample was analysed in triplicate, and a single RT(−) reaction performed as a negative control. Melt curve analysis of each run confirmed expected product sizes, and standard curves for each set of primers were derived using increasing dilutions of FT3010 fetal ovarian somatic cell cDNA to determine amplification efficiency. Threshold values (Ct) and gradients of standard curve slopes determined the concentration of each gene of interest in each test sample. Gene-of-interest expression was normalised to that of the housekeeping gene *RPL32*, which remained stable across gestation and between treatments.

### RT-PCR and agarose gel analysis of BMP receptors

Expression of BMP receptors in human fetal ovary and fetal ovary somatic cell cultures was assessed by end-point RT-PCR of RNA from fetal ovary RNA and RNA from 9, 15 and 17 week ovary somatic cell lines. RT- controls were also included. Reactions contained 500 nM each of each primer (Supplementary Table 1) and 1 µl of a 1/6 dilution of each cDNA (or water) in a 25 µl volume in 1× MyTaq HS Red Mastermix (Bioline, London, UK). 2-step reaction conditions were 95°C for 1 min. followed by 35 cycles of (95°C, 5 s/ 60°C, 20 s) in a PTC-100 Thermocycler (MJ Research). 10 µl of each reaction was electrophoresed alongside 100 bp DNA Ladder markers (Bioline) through a 2.5% agarose/ 1× Tris/Acetate/EDTA gel containing a 1/10 000 dilution of GelRed (Cambridge Biosciences, Cambridge, UK) and imaged under UV transillumination on a UGENIUS gel documentation system (Syngene Europe, Cambridge, UK).

### Protein extraction

Protein was extracted from fetal ovaries by homogenisation in 100 µl 1× RIPA Buffer (25 mM Tris.HCl, pH7.5; 150 mM NaCl ; 0.1% (v/v) SDS; 1% (v/v) Triton-X100; 0.05% (w/v) sodium deoxycholate and Mini Complete protease and phosphoSTOP phosphatase inhibitors (Roche)), using a Pellet Pestle (Sigma-Aldrich), or from cells in 6-well plates by washing twice in DPBS before lysis in 250 µl 1× RIPA Buffer and transferring the lysate into a 1.5 ml micro-centrifuge tube on ice. All lysates were spun at 14 000 *g* for 10 min at 4°C and the supernatants transferred to fresh tubes on ice. Protein concentrations were determined using the Bio-Rad DC Protein Assay (Bio-Rad Laboratories Ltd., Herts., UK).

### Western blotting and band quantification

Twenty µg (for GREM1) or 10 μg (for pSMAD1/5/8) of protein lysates were mixed 3:1 with 4× SDS sample buffer (250 mM Tris.HCl, pH6.8; 40% (v/v) Glycerol; 4% (w/v) SDS; 0.02% (w/v) Bromophenol Blue with 15% (v/v) 2-ME added just prior to use), denatured at 99°C for 6 min, then loaded alongside 5 µl of PageRuler Plus Prestained Protein Ladder (Fisher Scientific) on 12 well 4–20% Mini-Protean TGX gels, run in 1×Tris/Glycine/SDS buffer (both Bio-Rad). Gels were rinsed twice in water for 5 min, equilibrated for 10 min in Pierce 1× Methanol – free Western Blot Transfer Buffer (Fisher Scientific) then blotted onto Immobilon-FL PVDF membrane (Millipore UK Ltd., Watford, UK) using a Pierce Semi-dry Blotting Apparatus (Fisher Scientific) for 9 min at 25 V. Membranes were blocked in Rockland Fluorescent Blocking Buffer (Tebu-Bio Ltd, Peterborough, UK) diluted 1:1 in PBS containing 0.1% Tween20 (PBST) for an hour. Primary antibodies (Supplementary Table 2) were diluted as indicated in 1:1 blocking buffer: PBST, and then incubated with the blots at 4°C overnight with shaking. Blots were washed four times in PBST, for 5 min each, and incubated in the dark for 1 h with dilutions of Infrared Dye-labelled anti-rabbit and anti-mouse secondary antibodies as indicated in Supplementary Table 2. After washing twice each in PBST and then PBS, blots were imaged on a LiCor Odyssey Infrared Scanner, using Image Studio 5.0 Software. The pSMAD1/5/8 blot was quantified by drawing equal sized rectangles around individual bands and allowing the software to detect the total fluorescence signal minus background at the relevant wavelength. pSMAD1/5/8 signals were normalised to β-actin in the sample.

## Statistical analysis

Fetal ovary gene expression data were not normally distributed and so were analysed by Kruskal–Wallis Test with Dunn's Multiple Comparisons post-hoc test. QRT-PCR data on cell culture treatments, which showed a normal distribution, were analysed by one-way ANOVA with Tukey's Multiple Comparisons post-hoc test. All analyses were performed using GraphPad Prism 6.0 software.

## Results

### Expression of BMP antagonists during human fetal ovarian development.

**Figure 1:**
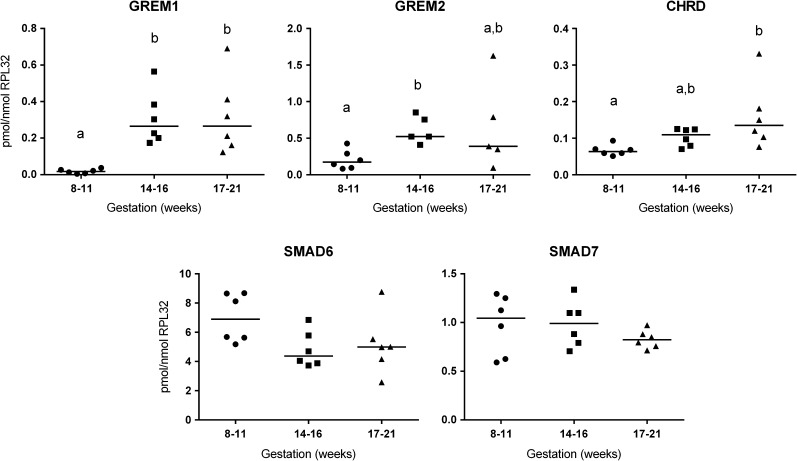
QRT-PCR Analysis of bone morphogenetic protein (BMP) antagonist expression across gestation in the human fetal ovary. Expression levels of each gene are given, normalised to the level of RPL32 in each sample. Ovary gestations are grouped to represent different stages in development as described in the main text. Results for individual samples (*n* = 5–6 for each stage) are presented along with median values. Changes between stages were analysed by the Kruskal–Wallis Test with Dunn's multiple comparisons and different letters above groups represent statistically significant differences (*p* ≤ 0.05, or less as indicated in the main text).

Expression of BMP antagonists *NOG*, *GREM1*, *GREM2*, *CHRD*, *SMAD6* and *SMAD7* was examined by qRT-PCR using cDNA samples corresponding to three stages of human fetal ovarian development, namely: 8–11 weeks (post-migratory germ cell proliferation), 14–16 weeks (entry of germ cells into meiosis) and 17–21 weeks (start of primordial follicle formation) (Fig. 1). Levels of *GREM1* mRNA increased 17-fold (*P* ≤ 0.05) between 8–11 and 14–16 weeks and this level was maintained at 17–21 weeks. *GREM2* expression increased 5-fold between 8–11 and 14–16 weeks gestation (*P* ≤ 0.05) and, as for *GREM1*, there was no further increase in *GREM2* between 14–16 and 17–21 weeks gestation. CHRD transcript levels increased more gradually, with expression rising 2.4 fold between 8–11 and 17–21 weeks (*P* ≤ 0.05). No significant change was detected in *SMAD6* or *SMAD7* levels across gestation. *NOG* transcripts were also detected (not shown), but at a level too low to permit accurate quantification.

### Human fetal ovarian somatic cells express somatic cell markers and components of the BMP signalling pathway *in vitro*

**Figure 2:**
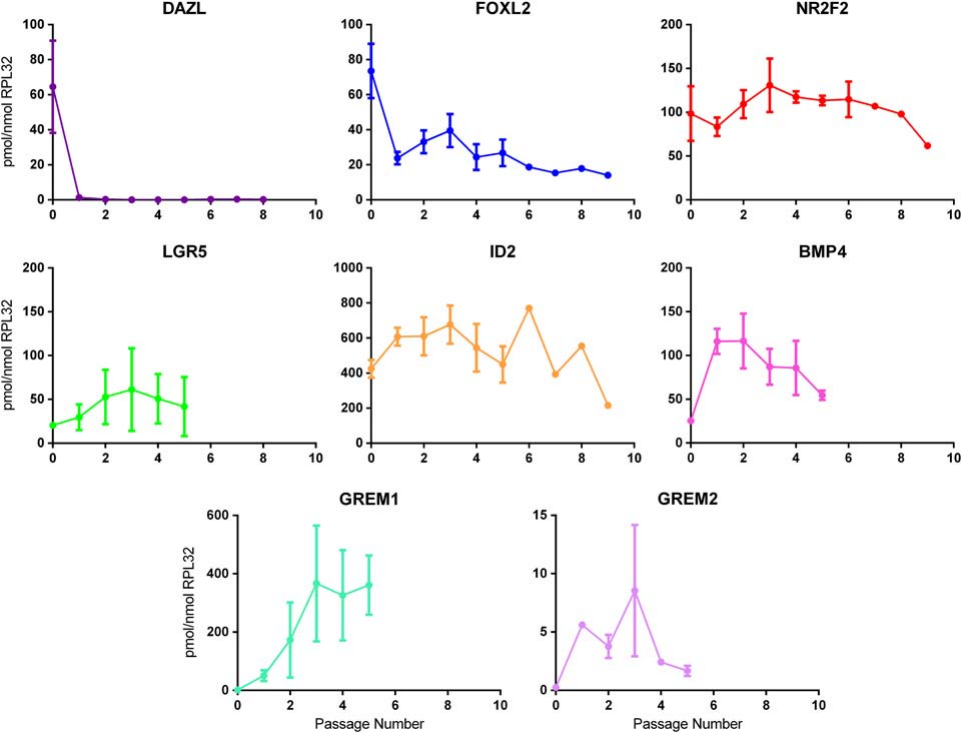
Expression of germ cell and various somatic cell markers with passage of adherent cells following disaggregation of human fetal ovaries. Expression of the germ cell marker *DAZL*, markers of differentiating pre-granulosa cells (*FOXL2*), steroidogenic cell precursors (*NR2F2*), immature dividing pre-granulosa cell precursors (*LGR5*) and components of the BMP pathway (*ID2*, *BMP4*, *GREM1* and *GREM2*) were examined by qRT-PCR across passages for a number of human fetal ovarian somatic cell cultures derived from 14 to 17 week disaggregated ovaries (*n* = 5 for *DAZL*, *FOXL2*, *NR2F2* and *ID2*; *n* = 4 for *BMP4*; *n* = 3 for *LGR5*, *GREM1* and *GREM2*). Results are expressed as mean expression relative to *RPL32* ± sem. The 0 time point represents disaggregated ovaries prior to plating.

To permit the functional analysis of BMPs and their antagonists in fetal ovary somatic cells, a number of primary ovarian somatic cell cultures (Table I) were derived from disaggregated human fetal ovaries by differential adhesion to cell culture plastic. The cultures had a fibroblast-like appearance and expanded continuously for at least 12 passages with a doubling time of 24–36 h. Passaging was necessary in order to derive sufficient cells on which to perform the experiments and to enable further experiments as data were obtained. Expression of ovarian somatic and germ cell markers was determined by qRT-PCR to assess changes with passage (Fig. 2). Expression of the germ cell marker *DAZL* was undetectable within two passages in each of the cell cultures, indicating germ cells were lost rapidly from the cultures (similar results were obtained with the germ cell markers *POU5F1* and *VASA*; data not shown). In contrast, expression of the somatic cell markers *FOXL2*, *NR2F2* (COUPTFII) and *LGR5* were retained with increasing passage number indicating that dedifferentiation was not occurring to a major extent. Levels of the BMP response gene *ID2*, which is expressed in somatic cells in human fetal ovary (unpublished data), remained fairly constant across passages in each of the cell cultures at least up to passage 5. BMP4 transcripts showed a decline with passage although remained readily detectable. *GREM1* expression tended to increase, and *GREM2* was also detectable throughout, although it was more variable. In summary, the cultures show significant phenotypic similarity and stability in their expression profiles, despite being derived from ovaries from fetuses of different gestational ages.

**Figure 3:**
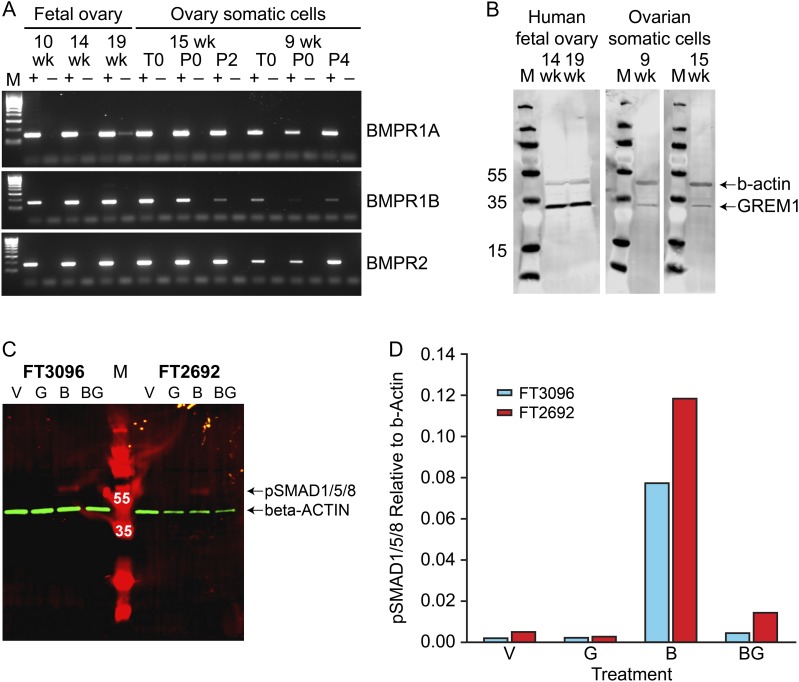
Components of the BMP signalling pathway are expressed in human fetal ovary and cultured ovarian somatic cells and regulate SMAD1/5/8 phosphorylation. **A**: Agarose gel analysis of RT-PCR products for the 3 BMP receptors (*BMPR1A*, *BMPR1B*, *BMPR2*). M is the 100 bp Hyperladder (Bioline). (+) and (−) represent reactions with RT(+) and RT(−) cDNAs respectively. Gestations of fetal ovaries and derived somatic cell cultures used are given in weeks (wk). T0 is cells from ovary disaggregation prior to plating (i.e. contains both germ and somatic cells), P0 is adherent somatic cells taken just prior to the first passage and P2-4 represents the number of passages undergone for each cell culture. **B**: Western blot analysis of GREM1 expression. M is the PageRuler Plus (10–250 kda) Prestained Protein Ladder (Fisher Scientific) and numbers beside individual marker bands give their MW as a guide. GREM1 (lower bands) with β-actin (upper bands) acting as a positive control. **C**: Western blot of protein extracts from cells (cultures FT3096 and FT2692) treated with vehicle (V), recombinant GREM1 (G), recombinant BMP4 (B) or BMP4 + GREM1 (BG) for 1 hour after overnight serum-starvation. M is the PageRuler Plus (10–250 kda) Prestained Protein Ladder (Fisher Scientific) and white numbers over individual bands indicate their MW. pSMAD1/5/8 signal (expected MW = 60) is red while the β-actin normalisation control (expected MW = 42) signal is green. **D**: Comparison of pSMAD1/5/8 levels normalised to β-actin signal for each treatment and cell culture (single replicates only performed).

We also determined expression of the BMP receptor genes (*BMPR1A*, *BMPR1B* and *BMPR2*) in fetal ovarian somatic cell cultures and across gestation in human fetal ovary by RT-PCR. Specific bands of the expected size were obtained for all three receptors (Fig. 3A). Expression of GREM1 protein in fetal ovary and ovarian somatic cells was demonstrated by western blotting, with anti-β-actin as a positive control for protein integrity. A band of the appropriate size (approximately 30 kDa) was detected in all ovary and somatic cell extracts (Fig. 3B).

### BMP signalling is functional in human fetal ovarian somatic cell lines and sensitive to GREM1

Expression of BMP receptors and antagonists in human fetal ovary somatic cell cultures suggested that the BMP pathway may be functional within these cells. To test this hypothesis, two independent somatic cell cultures (derived from 9 weeks (FT3096) and 15 weeks (FT2692) ovaries) were treated for 1 h with either (i) vehicle, (ii) 1 µg/ml recombinant human GREM1 (iii) 50 ng/ml recombinant human BMP4 or (iv) 50 ng/ml recombinant human BMP4 and 1 µg/ml recombinant human GREM1. Western blotting indicated that there was negligible pSMAD1/5/8 signal in extracts of vehicle or GREM1-treated cells from both the 9- and 15-week cultures, but BMP4 treatment for 1 h resulted in a 36-fold increase in pSMAD1/5/8 levels in FT3096 (9 weeks) and a 23-fold increase in FT2692 (15 weeks) cell lysates (Fig. 3C,D). This effect was reduced, but not completely abolished, when cells were treated with a combination of GREM1 and BMP4. These data indicate that human fetal ovarian somatic cells display the ability to receive and transduce BMP signals, and that this signalling can be antagonised by GREM1.

### BMPs 2 and 4 exert differential effects on gene expression in human fetal ovarian somatic cells

**Figure 4:**
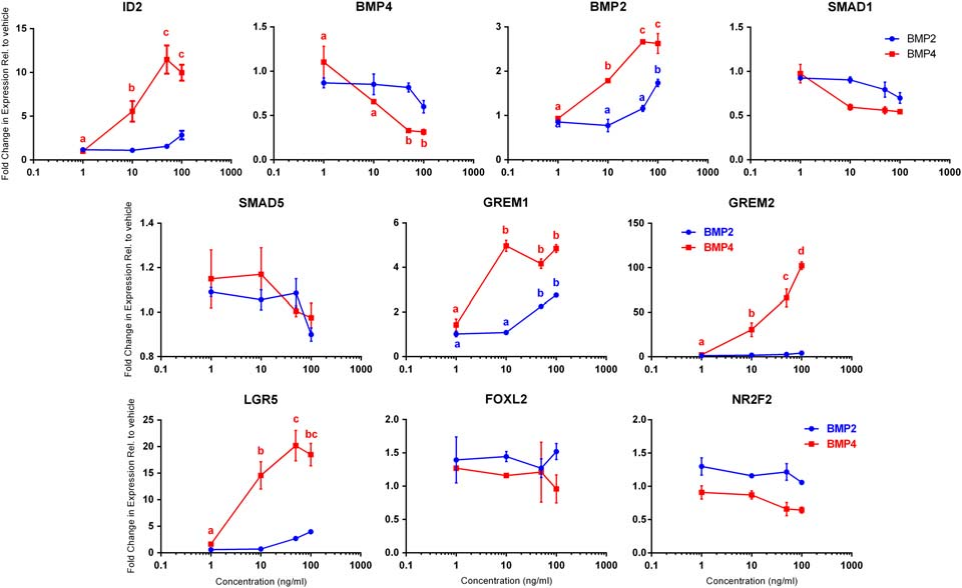
Titration of BMP4 and BMP2 effects on downstream gene expression in human fetal ovarian somatic cells. FT2692 ovarian somatic cells were serum-starved overnight and then treated (2 replicate wells per treatment) with vehicle or 1, 10, 50 or 100 ng/ml of recombinant human BMP4 or BMP2 for 24 h and RNA extracted. QRT-PCR for the genes indicated was performed and normalised to levels of *RPL32* before calculating fold changes relative to the vehicle control. Data points are mean ± sem. BMP2 treatment graphs are blue while BMP treatment graphs are red. Data were analysed by ANOVA with Tukey's multiple comparisons post-hoc tests and significant differences (*P* ≤ 0.05) between treatment concentrations are indicated by different letters (with colour matching key) above the relevant concentration.

In the human fetal ovary, *BMP4* expression decreases across gestation while *BMP2* mRNA levels increase, suggesting that these two BMPs might have different functional roles during ovarian development (Childs *et al*., 2010). This was investigated by comparing the effect of treatment with BMP2 or BMP4 on gene expression in human fetal ovarian somatic cells (FT2692 from 15-week ovary) (Fig. 4). ID genes are known immediate downstream targets of BMPs in other systems, and we observed an increase in *ID2* of up to 12-fold at concentrations ≥10 ng/ml of BMP4. Although there seemed to be a small increase in *ID2* at higher concentrations of BMP2, this did not reach significance. BMP4-treatment induced a decrease in *BMP4* gene expression (of up to 4-fold), although this was only significant at BMP4 concentrations ≥50 ng/ml, while BMP2 had no significant effect on *BMP4* gene expression. Treatment with either BMP2 (100 ng/ml) or BMP4 (≥10 ng/ml) resulted in significantly increased *BMP2* gene expression (up to 3-fold *P* < 0.05). Expression of *GREM1* increased in response to treatment with either BMP, rising 5-fold at ≥10 ng/ml BMP4 and over 2-fold at ≥50 ng/ml BMP2. *GREM2* expression was sensitive only to BMP4 treatment, rising up to 100-fold with BMP4, while BMP2 had no effect. There was no significant effect of either BMP on the expression of *SMAD1* or *SMAD5*.

We also examined the effect of BMPs 2 and 4 on the expression of key somatic cell markers in ovarian somatic cell cultures. Strikingly, we found that BMP4 treatment induced increases of up to 20-fold in the expression of *LGR5*, at concentrations ≥10 ng/ml (small increases in LGR5 expression at ≥50 ng/ml BMP2 were not significant). This identifies *LGR5* as a novel transcriptional target of BMP signalling in the developing human fetal ovary. Neither BMP2 nor 4 had any significant effect on expression of *FOXL2* or *NR2F2* transcript levels, indicating the effect on *LGR5* to be specific.

In view of this finding, changes in *LGR5* expression were investigated across gestation by qRT-PCR. *LGR5* mRNA levels fell from 57.8 ± 18.5 pmol/nmol *RPL32* at 8–11 weeks gestation to 12.0 ± 5.5 at 14–16 weeks, are remained at a similar level at 17–21 weeks (11.7 ± 5.9 pmol/nmol *RPL32*; *P* = 0.005, *n* = 6 per group).

### GREM1 and GREM2 antagonise the effects of BMP4 on transcription in a gene-specific manner

**Figure 5:**
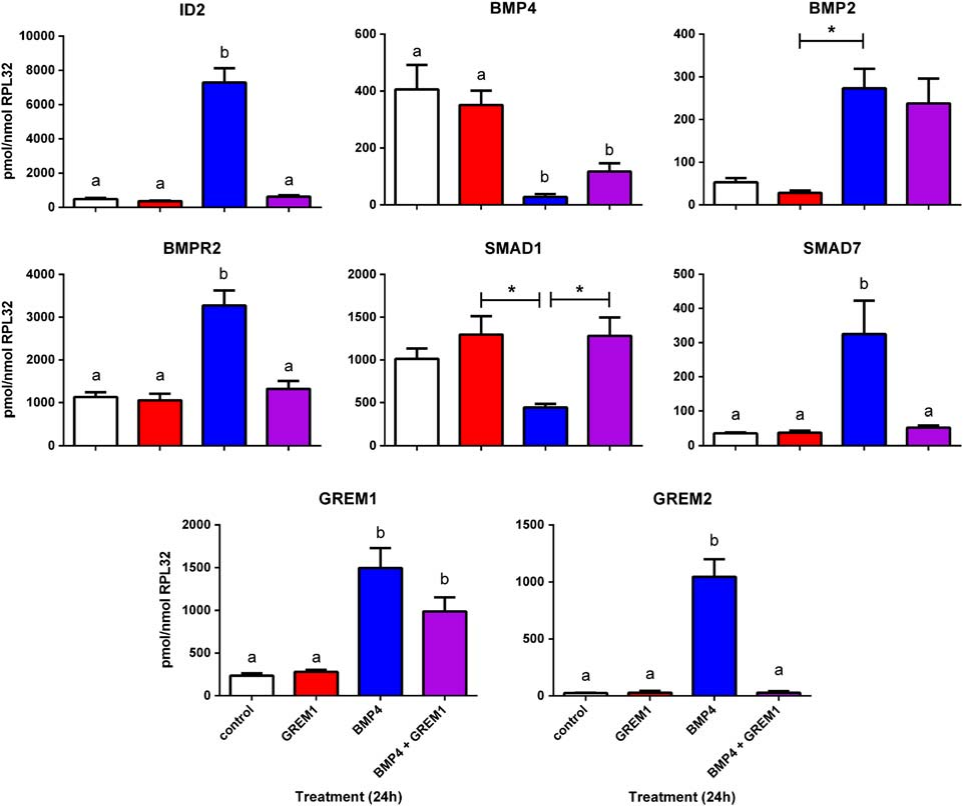
GREM1 sensitivity of BMP4 target genes in human fetal ovarian somatic cells. FT3010 cells were serum-starved overnight and treated with ± 1 µg/ml GREM1, ± 50ng/ml BMP4 (*n* = 4 replicate wells for each treatment) for 24 hours before RNA extraction and qRT-PCR analysis of key BMP4 targets, with normalisation to RPL32 transcript levels. Columns represent mean ± sem for each treatment (white = vehicle, red = GREM1, blue = BMP4, purple = BMP4 + GREM1). Differences in expression levels between treatments were analysed by ANOVA with Tukey's multiple comparisons post-hoc tests and significant differences (*P* ≤ 0.05 or less, as indicated in the main text) between treatments are indicated by different letters above the columns, or * in the case of *SMAD1* (*P* = 0.01).

Having demonstrated that GREM1 can inhibit phosphorylation of SMAD1/5/8 in response to BMP4, and that a number of genes expressed in ovarian somatic cells are BMP-sensitive, we next tested whether GREM1 could affect expression of genes regulated by BMP4, either alone or in combination with BMP4. Human fetal ovarian somatic cells were treated with vehicle, 1 µg/ml recombinant human GREM1, 50 ng/ml recombinant human BMP4 or both BMP4 and GREM1 in serum-free medium for 24 hours, and expression of key transcripts analysed by qRT-PCR (Fig. 5).

Treatment with GREM1 alone had no significant effect on any of the genes examined. BMP4 alone led to a 15-fold increase in expression of *ID2*, and this increase was abolished completely by the addition of GREM1. GREM1 reduced, but did not abolish, the inhibitory effect of BMP4 treatment on *BMP4* expression, but had no effect on the stimulation of *BMP2* expression by BMP4. The effects of BMP4 treatment on *BMPR2*, *SMAD1*, *SMAD7* and *GREM2* (either stimulatory or inhibitory) were also prevented by GREM1, whereas GREM1 did not inhibit the stimulation of expression of *GREM1* gene expression by BMP4. It was not possible to analyse properly the effects of treatments on *LGR5* expression in FT3010 cells as expression was so low in vehicle, GREM1 and BMP4 plus GREM1-treated cells that it was not reliably detectable although it was readily detectable in BMP4 treated FT3010 cells (data not shown), confirming our earlier result that *LGR5* is BMP4-responsive in culture FT2692 (Fig. 4). Since addition of GREM1 to BMP4 led to unreliable *LGR5* detection, this BMP4 effect also appears to be GREM1 sensitive.

**Figure 6:**
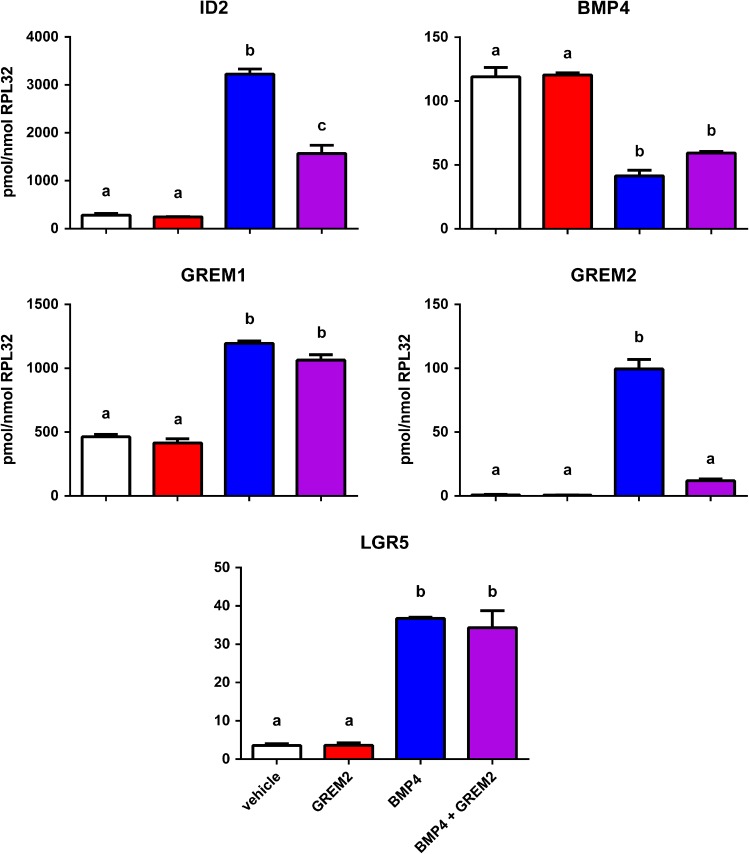
GREM2 Sensitivity of BMP4 Target Genes in human fetal ovarian somatic cells. FT3330 cells were serum-starved overnight and treated with ± 100 ng/ml GREM2, ± 50 ng/ml BMP4 (*n* = 4 replicate wells for each treatment) for 24 h before RNA extraction and qRT-PCR analysis of key BMP4 targets, with normalisation to *RPL32* transcript levels. Columns represent mean ± sem for each treatment (white = vehicle, red = GREM2, blue = BMP4, purple = BMP4 + GREM2). Differences in expression levels between treatments were analysed by ANOVA with Tukey's multiple comparisons post-hoc tests and significant differences (*P* ≤ 0.05 or less, as indicated in the main text) between treatments are indicated by different letters above the columns.

GREM2 is also expressed in human fetal ovary and ovarian somatic cells, and is up-regulated by BMP4, therefore we also examined possible interactions between recombinant GREM2 with BMP4 in an ovarian somatic cell line (Fig. 6). As with GREM1, addition of GREM2 alone had no effect on any genes examined, but displayed a similarly variable capacity to antagonise the effects of BMP4 on gene expression. GREM2 was effective at reducing the magnitude of the BMP4-induced increases in *ID2* and *GREM2* expression, but did not affect the respective decrease and increase of *BMP4* and *GREM1* expression in response to BMP4 treatment. The effect of GREM2 on the BMP4-induced stimulation of *LGR5* expression was also examined, but found to be ineffective (despite similarities in the level of BMP4-induced *LGR5* expression in the two cell cultures examined) which were generally higher for other induced genes in FT3010 cells than in FT3330, which is from a similar gestation.

## Discussion

BMP signalling can be regulated by a range of inhibitory factors, both intracellular and extracellular (Walsh *et al*., 2010), which provide mechanisms to limit the intensity and/or physical range of BMP signals. In the mouse, the BMP antagonist, GREM1 is required for the timely assembly of primordial follicles, indicating that restraining the intensity of BMP signalling is important for follicle formation to proceed (Myers *et al*., 2011). While BMPs are known to be expressed in the developing human ovary (Childs *et al*., 2010), the presence of their antagonists has not been examined. In this report, we investigated the expression of a number of BMP antagonists in the human fetal ovary, in cultures of ovarian somatic cells, and their functional ability to antagonise BMP-induced changes in gene expression. Human fetal ovarian somatic cells were shown to be more sensitive to BMP4 than BMP2, and GREM1 and GREM2 were shown to antagonise some but not all effects of BMP4 on these cells. This indicates the involvement of these regulatory pathways in human ovarian development, and that the modulation of BMP signalling by GREM1 and GREM2 is more complex than simply limiting it, but rather provides a mechanism whereby BMP responses become more selective.

The expression of BMP antagonists was demonstrated in both normal fetal ovary across a range of gestations, and in isolated somatic cells. Expression of the genes encoding the related extracellular antagonists GREM1, GREM2 and CHRD increased with gestation, suggesting that they have roles in BMP signalling during human ovary development in the lead-up to follicle formation. While these antagonists are best characterised for their interactions with BMP signalling, other functions for GREM1 have also been identified, including regulation of chemotaxis via Slit proteins (Chen *et al*., 2004) and by binding to heparin proteoglycans on the cell surface to enhance angiogenesis (Chiodelli *et al*., 2011). BMP/GREM interactions were further explored by isolating and characterising a number of primary human fetal ovarian somatic cell cultures, to allow functional studies not possible in organ culture. These cultures become rapidly devoid of germ cell markers, but they retain expression of the somatic cell markers *LGR5*, *FOXL2* and *NR2F2*. *LGR5* expression was quite variable, while *FOXL2* levels fell initially and then levelled off and *NR2F2* levels remained fairly constant in each cell line.

We have shown previously, by immunohistochemistry, that BMPR1A and BMPR1B, as well as pSMAD1/5/8 are predominantly expressed by germ cells in the human fetal ovary (Childs *et al*., 2010). Using RT-PCR, we have now demonstrated that BMP receptors are expressed not only in intact human fetal ovary but also in isolated ovarian somatic cells from the earliest passages and that these receptors function to promote BMP signalling through phosphorylation of SMAD1/5/8. While it is possible that expression of BMP receptors in the somatic cells was acquired or increased during culture, we have previously reported expression of BMPR1a in the somatic cells of the 9 weeks human fetal ovary (Childs *et al*., 2010), and it is possible that low-levels of BMP receptor or pSMAD1/5/8 expression in somatic cells at later developmental stages may have gone undetected due to the stronger immunohistochemical signal from these antigens seen in germ cells. We also demonstrate expression of a number of intracellular and secreted BMP antagonists in the fetal ovary which are transcribed in the somatic compartment. Germ cell expression of these antagonists is also possible, but remains unexplored.

BMP4 and, to a lesser extent BMP2, altered expression of genes involved in BMP signalling within the somatic compartment, including their own expression and two of their antagonists (GREM1 and GREM2), providing evidence for positive and negative feedback loops, and suggesting tight control of BMP signalling within the somatic compartment to maintain normal growth and development. This tight regulation is also supported by the demonstration that BMP4 inhibits its own transcription, while increasing expression of *BMP2*. BMP2 was markedly less effective at altering gene expression in the ovarian somatic cells, although this could, at least in part, be due to different biological activities of the recombinant proteins.

As the expression of *GREM1* and *GREM2* increased with gestation, we explored the interaction of these extracellular antagonists with the effects of BMP4 on ovarian somatic cells derived from ovaries of appropriate gestation. Confirmation of biological activity was provided by the demonstration that recombinant GREM1 reduced BMP4-mediated phosphorylation of SMAD1/5/8 in isolated ovarian somatic cells. Furthermore, GREM1 had a marked effect on the ability of BMP4 to alter expression of a number of genes (*ID2*, *BMPR2*, *SMAD1*, *SMAD7* and *GREM2*, and possibly also *LGR5*) expressed by ovarian somatic cells. GREM1 was able to prevent both stimulatory (*ID2*, *GREM2*) and inhibitory (e.g. *SMAD1*, *BMPR2*) effects of BMP4. Similarly, GREM2 was also able to inhibit the stimulatory effect of BMP4 on *ID2* and *GREM2* expression although genes which are inhibited with BMP4 were not tested for the effects of GREM2. However intriguingly, not all effects of BMP4 were prevented or reduced by GREM1 or GREM2; changes in the expression of *BMP4* and *GREM1* were not antagonised by GREM1/2 protein, and nor was BMP2 (at least in the presence of GREM1). Differential effects of GREM1 and GREM2 were demonstrated on BMP4 stimulated *LGR5* expression, which was insensitive to GREM2 but appeared to be sensitive to GREM1, although a lower concentration of GREM2 was used (using the manufacturer's recommended working concentrations). Overall, these results are in keeping with the suggested role of GREM1 in limiting BMP function during primordial follicle formation in the mouse ovary (Myers *et al*., 2011), but with a hierarchy of genes in terms of their sensitivity to blockage of BMP4 function in the presence of GREM1 or GREM2. It is possible that this reflects different affinities for pSMAD1/5/8 on different promoters when BMP4 activity is limiting, with GREM1 and BMP4 genes having the highest affinity for pSMAD1/5/8, in order to maintain the feedback loop and tight control of BMP signalling activity. This may serve to provide a mechanism of selectivity of BMP action on the somatic compartment of the developing ovary. In turn this may ‘fine-tune’ the functional activity of the BMP system in relation to other signalling pathways: crosstalk with TGFβ signalling is well described in several organs and tissues, notably in the regulation of tissue fibrosis (Yoshida *et al*., 2014) which may be of relevance to ovarian development and disease (Hatzirodos *et al*., 2011).

A striking finding of our study is the identification of *LGR5*, a marker of undifferentiated somatic cells, as a transcriptional target of BMP signalling in human fetal ovarian somatic cells. This positive regulation of *LGR5* by BMP4 was initially surprising given that suppression of BMP signalling (coupled with active WNT signalling) is required for the renewal of LGR5-positive intestinal crypt stem cells (Reynolds *et al*., 2014), while a switch from inactive WNT/active BMP towards active WNT/inactive BMP pathways in the Human Intestinal Epithelial Crypt (HIEC) cell line was sufficient to trigger a robust intestinal stem cell signature, including predominant LGR5 expression (Guezguez *et al*., 2014). However, our result is consistent with BMP4 expression being highest at early stages of ovarian development during the time of germ cell proliferation, with reduced expression from the time of onset of meiosis (Childs *et al*., 2010) when there is a need for more differentiated pre-granulosa cells to enable follicle formation to occur, and expression of *LGR5* showed parallel changes with increasing gestation. The absence of an effect of BMP treatment on the expression of *FOXL2* or *NR2F2*, markers of more differentiated pre-granulosa cells and stromal cells, respectively, is consistent with this. LGR5 is a G-protein coupled receptor which acts as a receptor for RSPONDIN to enhance RSPO/WNT/β-catenin signalling. In the mouse, cells expressing LGR5 lie within and immediately below the surface epithelium of the developing ovary, and it is believed that, as they proliferate, they ingress further into the ovary where they up-regulate Foxl2 and down-regulate Lgr5, thus differentiating into pre-granulosa cells (Rastetter *et al*., 2014). How this developmental transition is established and maintained remains unknown, but it seems likely that localised spatial differences in the repertoire or intensity of growth factor signals within different regions of the developing ovary may be involved. Similarly, in the human fetal ovary, a developmental gradient of germ cell differentiation is observed, with undifferentiated germ cells towards the periphery, and progressively more differentiated germ cells found more centrally, culminating with the formation of the first follicles deep within the ovary (Fulton *et al*., 2005). Such differences in the local growth factor microenviroment required to maintain this gradient would also impact on somatic cells, possibly influencing their phenotype or developmental fate depending on their location within the ovary. Establishing whether there are spatial differences in the expression of BMPs and their antagonists, LGR5, and FOXL2 within the fetal ovary may provide insight into this.

In conclusion, these data show the expression of BMP signalling antagonists in the human fetal ovary. Furthermore, we find that BMP signalling is active in human fetal ovarian somatic cells *in vitro*, and can be modulated by the extracellular antagonists GREM1 and GREM2. Differential effects of these antagonists may contribute to modulation of the effects of BMP signalling and provide a mechanism for fine-tuning of these effects on the somatic compartment of the ovary. Finally, we reveal that *LGR5* is a novel transcriptional target of BMP signalling in cultured ovarian somatic cells, suggesting possible roles for BMP signalling in regulating the development of the somatic cell compartment in the fetal ovary.

## Supplementary data

Supplementary data are available at http://molehr.oxfordjournals.org/.

Supplementary Data
