# Supplementary material for: BMP signalling in human fetal ovary somatic cells is modulated in a gene-specific fashion by GREM1 and GREM2
Source: Mol Hum Reprod. 2016 Sep 6;22(9):622–33. doi: 10.1093/molehr/gaw044 (PMC5013871; doi:10.1093/molehr/gaw044)
Supplement: Supplementary Data [file supp_22_9_622__index.html]

BMP signalling in human fetal ovary somatic cells is modulated in a gene-specific fashion by GREM1 and GREM2 — BMP signalling in human fetal ovary somatic cells is modulated in a gene-specific fashion by GREM1 and GREM2 — Supplementary Data 

# BMP signalling in human fetal ovary somatic cells is modulated in a gene-specific fashion by GREM1 and GREM2

## Supplementary Data

- Supplementary Data - docx file
