# Supplementary material for: BMP signalling in human fetal ovary somatic cells is modulated in a gene-specific fashion by GREM1 and GREM2
Source: Mol Hum Reprod. 2016 Sep 6;22(9):622–33. doi: 10.1093/molehr/gaw044 (PMC5013871; doi:10.1093/molehr/gaw044)
Supplement: Supplementary Data [file supp_gaw044_BMP_Antagonists_Paper_Suppl_Tables.docx]

**Supplementary Table I. RT-PCR Primers**

| **Gene** | **Forward Primer** | **Reverse Primer** | **Product Size** | **Genbank Acc. No.** | **Source** |
| --- | --- | --- | --- | --- | --- |
| GREM1 | CGTGTGAAGCAGTGTCGTTG | CTCATGCACACGAACTACGC | 200bp | NM_013372.6 | Primer BLAST |
| GREM2 | CGCGCTTCTCTTATGGGCG | AGCACCTCCTTGATCTGGTG | 172bp | NM_022469.33 | (Nio-Kobayashi *et al.* , 2015) |
| CHRD | CCCAGAGACCAGAACTGATCCA | GTGGTTCCCAGAGGTAGTGG | 205bp | NM_003741.2 | Primer BLAST |
| NOG | GCTGCGGAGGAAGTTACAGA | GAGCACGAGCGCTTACTGAA | 135bp | NM_005450.4 | Primer BLAST |
| SMAD6 | CCTACTCTCGGCTGTCTCCT | GAATTCACCCGGAGCAGTGA | 116bp | NM_005585.4 | Primer BLAST |
| SMAD7 | CTGTCCAGATGCTGTGCCTT | AATTGAGCTGTCCGAGGCAA | 236bp | NM_005904.3 | Primer BLAST |
| BMPR1A | GATGGTCCGGCAAGTTGGTA | TGGCGCATTAGCACAGTTTG | 150bp | NM_004329.2 | Primer BLAST |
| BMPR1B | GAGGATGACTCTGGGTTGCC | AGGCAGTGTAGGGTGTAGGT | 159bp | NM_001256793.1 | Primer BLAST |
| BMPR2 | GTTTCTCCGCCGGTCTACTT | ACGCACATAGCCGTTCTTGA | 172bp | NM_001204.6 | Primer BLAST |
| BMP4 | TGAGCCTTTCCAGCAAGTTT | CTTCCCCGTCTCAGGTATCA | 180bp | NM_001202.4 | (Childs *et al.*, 2010) |
| BMP2 | GTTCGGCCTGAAACAGAGAC | GAATCTCCGGGTTGTTTTCC | 217bp | NM_001200.3 | (Childs *et al.*, 2010) |
| SMAD1 | CTACCCTCACTCTCCCACCA | GCACCAGTGTTTTGGTTCCT | 211bp | NM_005900.2 | (Childs *et al.*, 2010) |
| SMAD5 | TTCTGGCTCAATCTGTCAACC | GGAGCCCATCTGAGTAAGGAC | 197bp | NM_005903.6 | (Childs *et al.*, 2010) |
| ID2 | AGGCTTCTGAATTCCCTTCTG | CCTCCTTGTGAAATGGTTGAA | 166bp | NM_002166.4 | Primer 3 |
| INHBA | AAGTCGGGGAGAACGGGTATGTGG | TCTTCCTGGCTGTTCCTGACTCG | 123bp | NM_002192.2 | Primer BLAST |
| LGR5 | CCCACACACTGTCATTGCGA | CGTTTCCCGCAAGACGTAAC | 199bp | NM_003741.2 | Primer BLAST |
| FOXL2 | TACTCGTACGTGGCGCTCAT | CTCGTTGAGGCTGAGGTTGT | 162bp | NM_023067.3 | Primer BLAST |
| NR2F2 | GCCATAGTCCTGTTCACCT | GCACACTGAGACTTTTCCTG | 83bp | NM_021005.3 | (Hubert *et al.* , 2010) |
| RPL32 | CATCTCCTTCTCGGCATCA | AACCCTGTTGTCAATGCCTC | 152bp | NM_000994.3 | (Zhang *et al.* , 2005) |

**Supplementary Table II. Antibodies for Western Blotting**

| **Antibody** | **Source** | **Working Dilution** | **Antibody Type** |
| --- | --- | --- | --- |
| Polyclonal Rabbit anti-GREMLIN (human) | Life Technologies  PA5-13123 | 1:1000 | Primary |
| Polyclonal Rabbit anti-pSMAD1/5/8 | Cell Signaling Technology #9511 | 1:1000 | Primary |
| Monoclonal Mouse anti-β-actin | Sigma-Aldrich  clone AC-15, A5441 | 1:5000 | Primary |
| Alexa680 Donkey anti-Rabbit IgG | Life Technologies  A10043 | 1:10,000 | Secondary |
| IRDye800 Donkey anti-Mouse IgG | Tebu-Bio, Rockland  610-732-124 | 1:10,000 | Secondary |
